# Supplementary material for: Observation of localized modes at effective gauge field interface in synthetic mesh lattice
Source: Sci Rep. 2019 Mar 5;9:3464. doi: 10.1038/s41598-019-39916-6 (PMC6401141; doi:10.1038/s41598-019-39916-6)
Supplement: Supplementary file 1 — Supplemental material: Observation of localized modes at effective gauge field interface in synthetic mesh lattice [file 41598_2019_39916_MOESM1_ESM.pdf]

Supplemental material: Observation of localized modes at effective gauge field interface in synthetic mesh lattice

Artem V. Pankov,[1] Ilya D. Vatnik,[1] Dmitry V. Churkin,[1] and Andrey A. Sukhorukov[2]

[1]*Novosibirsk State University, Pirogova str. 2, Novosibirsk 630090, Russia*

[2]*Nonlinear Physics Centre, Research School of Physics and Engineering, Australian National University, Canberra, ACT 2601, Australia*

1. EXPERIMENTAL SETUP

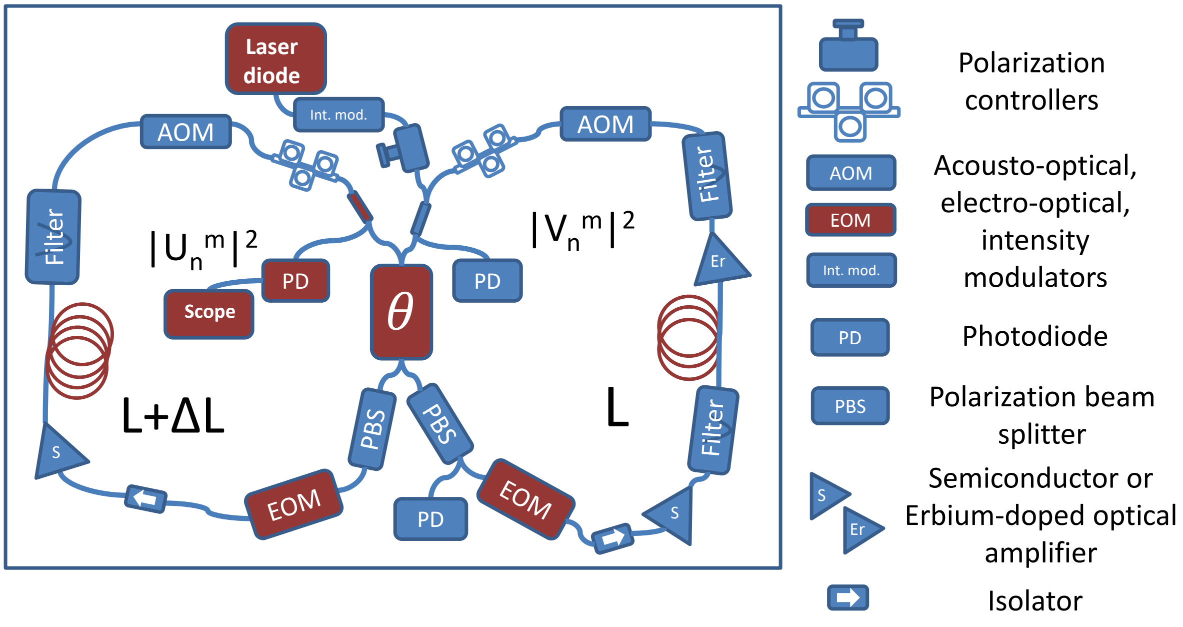

FIG. S1. Detailed experimental setup.

The setup used in the experiment is similar to one described in [1]. The synthetic photonic mesh lattice was built up with a pair of 5 km of non-zero dispersion-shifted telecommunication fiber spools. There was a small difference of 75 m in lengths of spools. Initial single pulse launched into the shorter loop was formed from a radiation of an FBG-stabilized diode laser by means of an intensity modulator (see Fig. S1). Width of the pulse was set to 60 ns and its central wavelength was 1550 nm. Optical losses of all elements were compensated using semiconductor and erbium doped optical amplifiers together with optical filters and optical isolators to suppress amplified spontaneous emission from amplifiers. In order to suppress noise circulating within loops losses per roundtrip were considerably increased before each measurement by means of acousto-optic modulators and set back at the very moment of the launching the first pulse into the system. As pulse polarization is altered by fiber random birefringence and amplifiers having polarization depended gain, it was compensated by several polarization controllers. Polarization state was monitored using a pair of polarization beam splitters. Two electro-optical phase modulators (EOM) were added into each loop in order to alter phase accumulation over a roundtrips. Phase was shifted

for all pulses placed into time slots with n≥1 to create a gauge field jump at $n = 0$. A photodiode detecting a pulse train was placed in the longer loop. Experiment has been done with three configurations: with a 50/50 coupler, and a 90/10 coupler with 10% port leading to the long or the short loop, that correspond to $\theta = \pi/4, 2\pi/5, \pi/10$ accordingly.

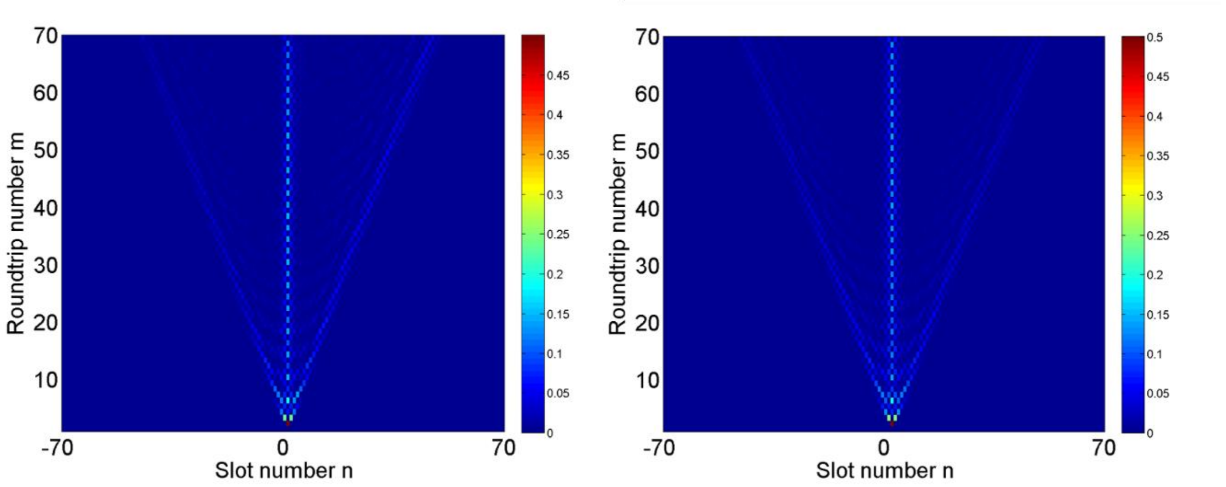

FIG. S2. Left panel - experimentally observed light evolution for the synthetic photonic mesh lattice with $\phi_0 = -\psi_0 = \pi/4$ and $\theta = 0.25\pi$. Right panel - numerically obtained evolution with the same parameters.

To study localized modes in a range of lattice parameters (Fig. 4 (a-c) of the Main Text) phase modulation amplitudes (ϕ_0, ψ_0) were varied in a range of $[0, 11\pi/8]$ which was defined by the capabilities of electrical generators driving the modulators. Note that there is a symmetry of the solutions of Eq. (3) from Main Text with phases (ϕ_0, ψ_0) and $(2\pi - \phi_0, 2\pi - \psi_0)$. This allowed us to extrapolate data found from the experiment over the most of the range [0, 2π] of phase shifts parameters (ϕ_0, ψ_0).

We observed that the measured light evolution is in excellent agreement with predictions of the straightforward numerical simulations of the Eq. (1) from Main Text with appropriate boundary and initial conditions, see Fig. S2.

2. DIFFERENT INITIAL EXCITATION POSITIONS

To emphasize the strong confinement of the localized modes intensity profiles $|u_n|^2$, we performed a series of experiments with gauge field interfaces moved to different slots n, while

the initial pulse was launched into the same slot $n = 0$ into the short loop. In full agreement with estimations of the inverse full width at half-maximum (IFWHM), localized modes were efficiently excited when the interface was placed in the vicinity δn$\sim$2 of the initial pulse position $n = 0$ (see Fig. S3).

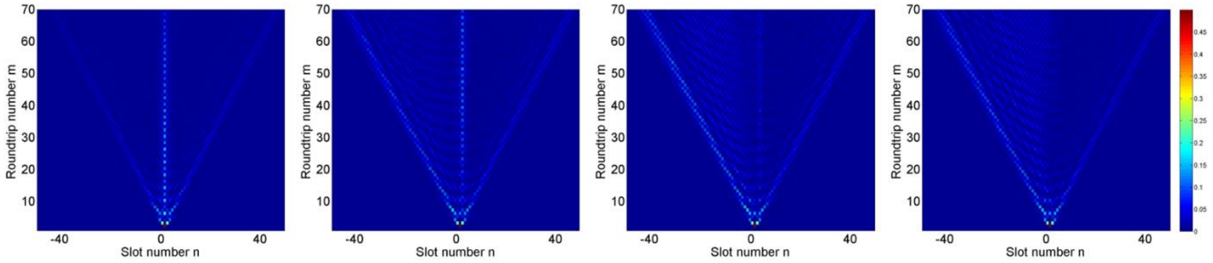

FIG. S3. Experimentally measured evolution of light in the synthetic photonic mesh lattice with gauge field interface realized in different slot positions. Initial pulse was launched into the same initial position $n = 0$. The interface is placed into a slot with $n = 0, 1, 2, 3$ for graphs from left to right. Here $\phi_0 = -\psi_0 = \pi/4$, $\theta = \pi/4$.

3. EXTRACTING LOCALIZATION WIDTH OF INTERFACE MODES

When a single pulse is launched into the synthetic photonic lattice, both non-localized and localized modes are excited. After a sufficient number of roundtrips, only two localized modes remain at the interface at $n = 0$ with different amplitude distributions u_n and $u_n^{'}$ but the same intensity distributions $|u_n|^2, |u_n^{'}|^2$ (see Fig. S4). These two modes have different propagation constants β and $\beta' = \beta - \pi$, according to Eq. (5) in the Main Text. Because of that, in the experiment we see oscillation of the sum of the modes with the period of $\Delta m = 2$. To obtain localized modes' intensity distribution from the measured total intensity I_n^m, we solve the following set of equations:

$$\begin{aligned} |u_n + u_n^{'}|^2 &= I_m, \\ |u_n - u_n^{'}|^2 &= I_{m+1}. \end{aligned} \tag{1}$$

and obtain

$$|u_n^2| = (I_n^m + I_n^{m+1})/4. \tag{2}$$

To calculate the inverse full width at half-maximum (IFWHM) of the intensity distribution $|u_n|^2$, we further fit $|u_n|^2$ on the left-hand and right-hand side vicinity of the inter-

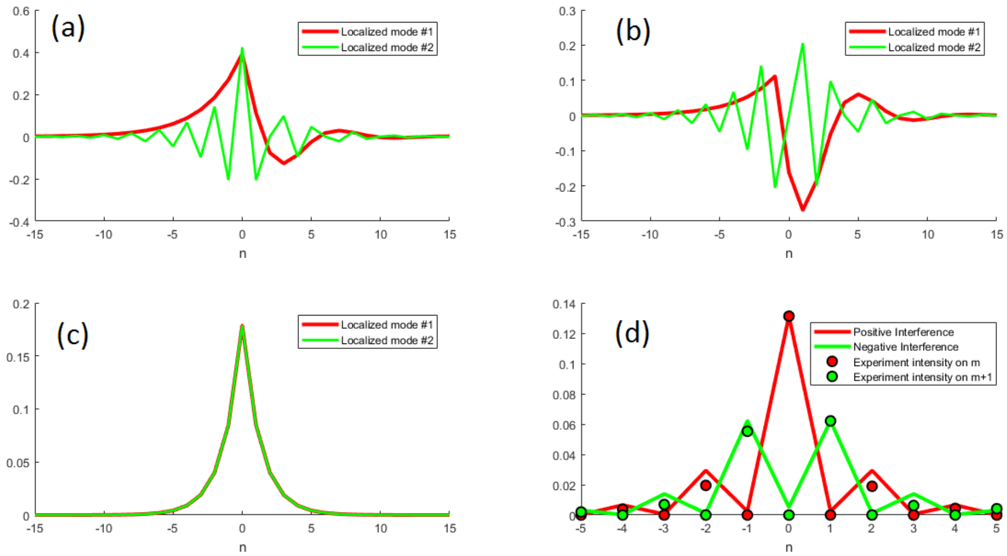

FIG. S4. (a) Real and (b) imaginary part of distributions $u_n, u_n^{'}$ for a pair of modes localized at $n = 0$. (c) Intensity distributions $|u_n|^2$, $|u_n^{'}|^2$ for the pair of modes localized at $n = 0$. (d) Results of interference of two modes at odd and even steps (m), $|u_n + u_n^{'}|^2$ and $|u_n - u_n^{'}|^2$, and corresponding experimentally measured data. Here $\phi_0 = -\psi_0 = \pi/4, \theta = \pi/4$.

face with exponential functions $\exp(k_l n)$ and $\exp(-k_r n)$, respectively, and find IFWHM $= (k_l k_r)/[(k_l + k_r)\ln 2]$.

4. EXPERIMENTAL ERRORS

In general, possible origins of experimental errors are the photodiode and oscilloscope technical noise, amplifier noise, errors in determining the lattice parameters (modulation depths etc), group velocity dispersion, polarization dependent dispersion and residual birefringence of the fiber, inexact balance between the total gain and losses. The noise of the measuring system manifests in the stochastic errors, when intensity in each slot n is calculated by averaging within the corresponding time-bin of the temporal dynamics measured by an oscilloscope (see Fig. S5). We calculate the standard errors for time-bin averaged intensities, and find that their values are at the level of 9% or lower, depending on roundtrip number. We also identify the main sources of systematic errors in our experiment: (i) an inexact balance of total gain and losses and (ii) the spontaneous noise of the amplifier. These

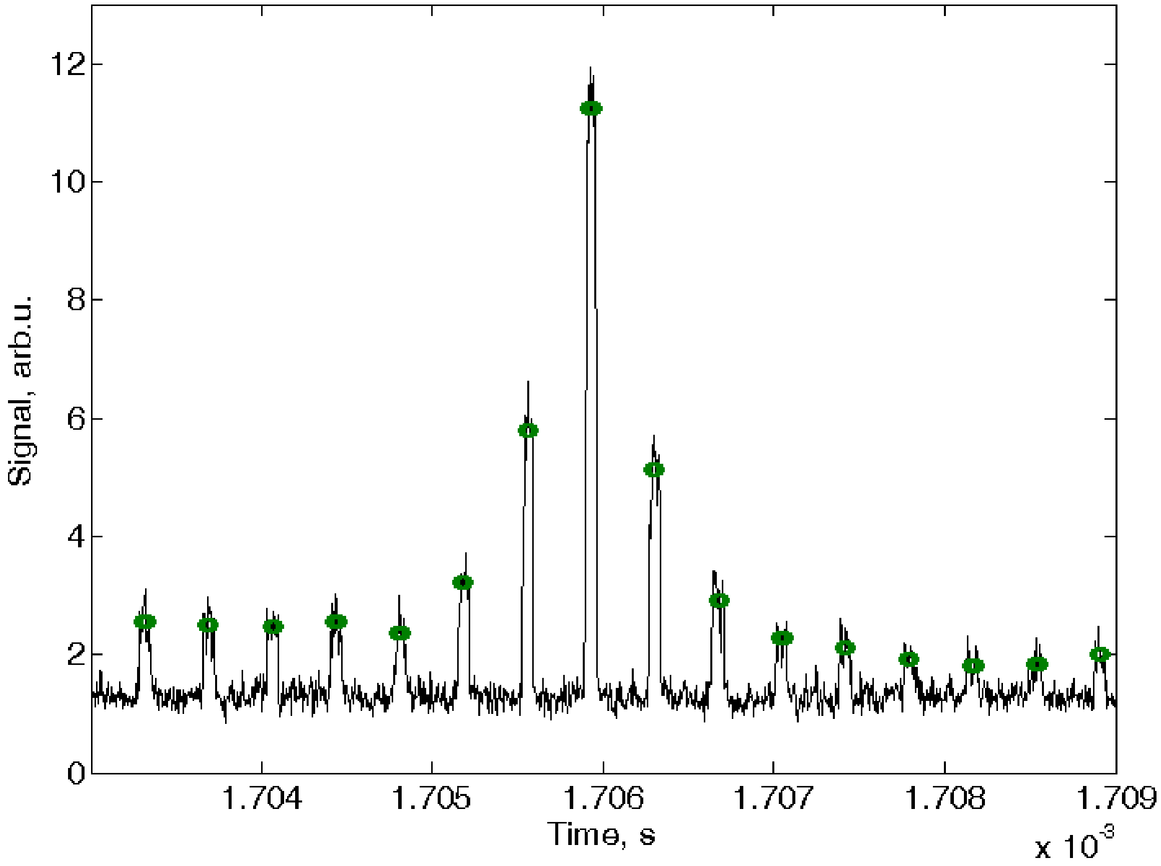

FIG. S5. Experimentally measured temporal dynamics of the pulse train at the roundtrip $m = 70$. Parameters correspond to the regime of mode localization. Black line is the signal from the oscilloscope, green dots – intensity averaged over the time-bins.

may alter the observation of localized modes, and we estimate an associated additional 5% of mode width uncertainty. We see that overall, the experimental noise has a small effect on the localized modes, which further demonstrates their robustness to perturbations.

---

[1] I. D. Vatnik, A. Tikan, G. Onishchukov, D. V. Churkin, and A. A. Sukhorukov, Sci. Rep. **7**, 4301 (2017).
